# Analysis of Changes in the Microstructure of Geopolymer Mortar after Exposure to High Temperatures

**DOI:** 10.3390/ma13194263

**Published:** 2020-09-24

**Authors:** Marta Dudek, Mateusz Sitarz

**Affiliations:** Chair of Building Materials Engineering, Faculty of Civil Engineering, Cracow University of Technology, 24 Warszawska St. 31-155 Cracow, Poland

**Keywords:** fly ash geopolymers, GGBFS, high-temperature exposure, porosity MIP, SEM, microstructure

## Abstract

The inorganic structure formed at the stage of setting of the geopolymer binder ensures high durability of the material under high-temperature conditions. However, changes in the microstructure of the material are observed. The purpose of the study was to analyze changes in the structure of geopolymer mortar after exposure to high temperatures T = 200, 400, 600, 800, and 1000 °C. Mortars with a binder based solely on fly ash (FA) and mixed in the 1:1 ratio with a binder containing fly ash and ground granulated blast-furnace slag (GGBFS) were tested. The descriptions of their microstructures were prepared based on digital microscope observations, scanning electron microscope (SEM) observations, EDS (energy dispersive spectroscopy) analysis, and mercury intrusion porosimetry (MIP) porosity test results. Changes in the material due to high temperature were observed. The differences in the microstructure of the samples are also visible in the materials that were not exposed to temperature, which was influenced by the composition of the materials. Porosity increases with increasing annealing temperature. The distribution of individual pores also changes. In both materials, the proportion of pores larger than 1000 nm increases with the temperature increase. Moreover, the number of cracks and their width also increases, reaching 20 µm in the case of GGBFS. Furthermore, the color of geopolymers has changed. The obtained results extend the current state of knowledge in the field of changes in the microstructure of geopolymers subjected to high temperature.

## 1. Introduction

The huge consumption of Ordinary Portland Cement (OPC) and traditional cementitious materials requires the development of alternatives in the construction sector. The problem is also the significant energy consumption and greenhouse gas emission associating the cement production processes. It is estimated that the whole process of producing 1 ton of cement consumes about 3.2 GJ of energy and generates nearly 810 kg of carbon dioxide (CO_2_), 1.0 kg of sulfur dioxide (SO_2_), and 2.0 kg of nitrogen oxides (NO_x_) [1]. Therefore, the development of sustainable bonding materials is important. In recent years, we have observed an increasing interest in geopolymer materials. Geopolymers have been paid more attention by researchers and industry. A great amount of research focuses on the possibility of using for the synthesis of geopolymers fly ash [2], red mud [3], biomass ash [4], heavy metal waste [5], and recycled glass [6]. Geopolymer materials are gaining ground due to their excellent properties and sustainable. The greatest advantages of geopolymers are strong bonding and good durability, high-temperature resistance, low permeability, great chemical corrosion resistance, and environmental friendliness [1]. Potential fields of application of geopolymer materials include geopolymer concrete, precast construction elements, materials on pavement, repair materials, protective coatings, immobilization of toxic metals, nuclear waste management, and three-dimensional printing materials [7,8,9,10].

A geopolymer is an inorganic mineral binder made from aluminosilicate raw materials, setting in an alkaline environment. Industrial waste, in particular fly ash and blast-furnace slag, can be used in the synthesis of geopolymers [11]. Of all waste materials, the most commonly used are low-calcium silica fly ash (FA) class F and ground granulated blast-furnace slag (GGBFS) [12,13]. Although the setting reaction mechanisms for both raw materials are similar, the reaction products are different [14,15]. In the case of a fly ash-based geopolymer, the main product of the reaction is amorphous hydrated alkali-aluminosilicate [11,15]. In the case of alkaline activation of GGBFS, the C-S-H phase is formed [16,17]. Recent studies on alkali-activated materials in CaO-SiO_2_-Al_2_O_3_ system characterize the hydration products as C-(N-)A-S-H. The C-(N-)A-S-H phase is formed over a wide range of CaO/SiO_2_ ratios. The most intensive development of the C-(N-) A-S-H structure is observed at a CaO/SiO_2_ ratio close to 1.0 and relatively low Al regions [18].

The basic group of factors affecting the structure of the geopolymer composite are the type of mineral raw materials used and the type of alkaline solution used. One of the methods of structure modification and strengthening of mechanical properties of the fly ash-based geopolymer is application of a mineral additive rich in calcium. Ground granulated blast-furnace slag is most frequently used for this purpose. Test results [19] indicate that the addition of slag in the amount of only 4% improves the strength of the material. Using a mixed FA-GGBFS binder in the 1:1 ratio, it is possible to obtain a mortar with compressive strength above 60 MPa after 28 days of curing without additional temperature treatment at the material setting stage [20]. The partial replacement of fly ash by ground granulated blast-furnace slag strengthens the structure of the material and can also accelerate the curing of the geopolymer at room temperature. The addition of GGBFS increases the calcium content in the material structure, increases early strength, shortens the setting time at ambient temperature and increases the density and mechanical parameters of the binder [21]. In the case of certain geopolymer composites with a mixed FA-GGBFS binder, high tensile stress caused by shrinkage was observed, which increases the risk of cracks in the material at the early stage of curing [22]. In the case of geopolymer materials, the microstructure and mechanisms of the formation of a contact zone between the geopolymer binder and aggregate are still not fully understood. It is believed that the cracks observed in the geopolymer binder with the addition of GGBFS may be caused by a change of volume occurring during the formation of amorphous to semi-crystalline CSH gel in a partially cured geopolymer gel [23]. Peng et al. [24] point out the existence of a transition zone between the geopolymer matrix and aggregate, formed as a result of a chemical reaction between an alkaline activator and chemical compounds derived from the aggregate. The contact zone differs in composition from the geopolymer matrix. Shrinkage, probably of the CSH gel, can also lead to cracks in the transition zone. In addition, a higher liquid/binder ratio in the mix leads to a very thin contact zone and promotes the formation of wider cracks. When curing a geopolymer binder with a high liquid/solid ratio, increased water evaporation occurs, which directly results in high shrinkage of the binder. 

Another possibility of introducing calcium into the structure of the geopolymer is the use of class C fly ash. However, the use of fly ash with a high calcium content (class C) does not bring the desired results. This means that Ca in fly ash class C does not dissolve in an alkaline solution as easily as Ca from GGBFS. Unlike calcium from GGBFS, calcium from fly ash does not improve the mechanical strength of the material. The calcium contained in GGBFS is able to form the C-S-H phase in contrast to the Ca contained in the class C fly ash, whose contribution to the form of geopolymer synthesis reaction products is minor. The difference in the availability of Ca for the production of geopolymeric structures results from different chemical forms of calcium in both compared raw materials [25]. 

The second group of factors influencing the structure of the geopolymer are the temperature treatment conditions applied at the material setting stage. The geopolymer binder sets in a wide range of temperatures. The results presented in the literature usually concern geopolymer materials setting at temperatures ranging from 20 °C to 90 °C [15]. Amorphous structures dominate in the geopolymer cured at room temperatures. The use of additional annealing in the material setting stage can lead to the formation of crystalline phases. Geopolymers based only on fly ash require temperature treatment during the curing stage in order to ensure an adequate rate of hardening and strength increase elevated temperatures during the curing and setting process increase the intensity of the polymerization reaction [15,26]. 

The influence of elevated temperature on the geopolymer binder can be analyzed in two aspects. As a type of temperature treatment during the curing and setting stage of the material and as a type of external load acting on the cured material. The further part of the literature review focuses on the factors influencing the behavior of the geopolymer under thermal load. A comprehensive model of evaluation of the influence of high temperature on the geopolymer binder assumes carrying out the analysis at three levels: micro scale (chemical stability), meso scale (deformation resistance), and macro scale (strength endurance, spalling resistance) [27]. The article focuses on the changes occurring on the micro scale. The knowledge of the transformations taking place in the microstructure of the geopolymer explains the behavior of the material under high-temperature conditions. 

The effect of high temperature on the microstructure of the geopolymer is clearly observed. High-temperature sintering and crystallization processes change the morphology of the geopolymer binder. The gradual process of water migration affects the structure and distribution of pores. All the changes occurring at the microstructure level influence the behavior of the material on the macro scale and determine the mechanical properties of the binder [27]. 

The first changes in the microstructure of the material in relation to samples not subjected to temperature exposure are already observed at temperatures up to approx. 200 °C and are caused by the removal of free and poorly bound water. An increase in temperature inside the material leads to the formation of water vapor. The process of intensive water removal from the material begins. As a result of increasing intrapore pressure, microcracks, and changes in the pore structure occur. The extent of observed microstructural changes depends on several factors. One of the parameters of key importance is the amount of water introduced at the mix preparation stage. The water content depends on the water demand of the precursor used and takes into account the workability requirements of the mix. Kong et al. [28] observed higher levels of damage in metakaolin-based geopolymers compared to materials based on fly ash. It was found that the differences were caused by a much higher amount of water required for the preparation of mixes with metakaolin and weaker binding of water molecules in the material structure. From the temperature of approx. 550 °C, changes in the microstructure of the geopolymer are caused by sintering and thickening processes in the matrix [29,30]. The results of conducted observations show less cavities and smoother texture of the geopolymer paste. Viscous sintering observed at high temperature can lead to healing of microcracks. This type of effect has been described in the literature [31]. In the case of a geopolymer composite in which aggregate is also used, the difference in thermal expansion coefficients between the binder and aggregate will be an additional factor affecting the microstructure of the material. Cracks in the binder matrix, in the contact zone between the matrix and the aggregate and cracks passing through the aggregate grains are observed in samples after exposure to high temperatures [32]. The level of aggregate damage depends on its chemical composition. Some silica aggregates can decompose at 350 °C [33]. Bakharev [30], when studying the behavior of the geopolymer with fly ash at 800 °C and 1000 °C, observed an increase in both average pore size and cumulative pore volume. According to the author, the increase in porosity was a result of crystallization processes taking place in the analyzed temperature range. Gourley et al. [34], when comparing geopolymer and cement binders, note the internal structure of both materials. The total amount of water contained in the pore structure of the geopolymer binder is relatively low and open porosity prevails in the material. Degradation of the material at a temperature of approx. 1000 °C progresses as a result of melting and is not explosive. Up to a certain temperature level, the water contained in the pores absorbs the heat supplied from outside, which effectively lowers the temperature of the sample and reduces the level of damage in the initial period. In the higher temperature range, the open pore structure allows water to migrate freely, limiting structural damage to the material. Rickard et al. [35] compared the effect of high temperature on the microstructure of two mixes with fly ash differing in density and initial strength. The material with a more compact structure was characterized by a higher level of damage and deformation and a clear decrease in strength. The material with a less compact structure demonstrated much better behavior under high-temperature conditions. The level of damage observed was significantly lower. Less compact internal structure of the material allowed for better compensation of changes in the material structure. After exposure to high temperatures, an increase in strength was visible due to sintering in the material.

The aim of the research was to analyze changes in the microstructure of geopolymer mortar after exposure to high temperatures. Materials cured at ambient temperature were tested and then subjected to external load in the form of temperature −200 °C, 400 °C, 600 °C, 800 °C and 1000 °C. Observations were made on samples cooled down to room temperature. Two mortars were analyzed, one based solely on fly ash, the other with the addition of ground granulated blast-furnace slag. The characteristics of microstructures were developed based on observations under the scanning electron microscope (SEM) and digital microscope. The changes occurring in the matrix, inclusions, and contact zone were analyzed. An EDS analysis was also performed to identify the elements that make up the material. The observations of the structure of the material were complemented by mercury intrusion porosimetry (MIP) testing.

## 2. Materials and Methods 

FA is a mineral additive commonly used in concrete technology and very often a base precursor for geopolymer binders. The fly ash used is characterized by the content of SiO_2_ > 40 wt.%, Al_2_O_3_ < 30 wt.%, CaO < 10 wt.%. According to the Polish classification of fly ash to its chemical composition (standard BN-79/6722-09) belongs to the group of siliceous fly ash. It is characterized by high content of SiO_2_ (52 wt.%) and Al_2_O_3_ (28 wt.%) with low CaO content (3 wt.%). The full oxide composition of fly ash is presented in Table 1. According to ASTM C618, it is class F ash. In addition, it meets the requirements of EN 450-1 and can be used for concrete production. Due to the roasting losses, it belongs to category A. The unburned carbon content does not exceed 5%. The ash density is 2.1 g/cm^3^. 

GGBFS from the Ekocem milling plant belonging to the Górażdże group was also used to prepare the geopolymer binder. The oxide composition of GGBFS is presented in Table 1. The main chemical components of GGBFS are CaO (43 wt.%) and SiO_2_ (39 wt.%). The GGBFS density is 2.9 g/cm^3^. Quartz sand in the 0/2mm grain size with a density of 2.65 g/cm^3^ was used for sample preparation. Quartz have good thermal stability. In the case of quartz aggregates, the most important changes take place at the temperature of 573 °C, where the low-temperature β-quartz is converted into the high-temperature α-quartz. The change leads to an increase in volume.

The alkaline solution used in the process of geopolymer binder synthesis contains an aqueous solution of sodium silicate Geosil^®^ 34417 and additionally introduced amount of water. The Geosil 34417 solution used has a molar ratio of MR = 1.7. The molar ratio, is the basic characteristic of aqueous solutions of sodium or potassium silicate equal to the ratio of the number of moles of silicon oxide to moles of metal oxide. Table 2 below presents the chemical composition and basic parameters of Geosil 34417 according to the technical card provided by the manufacturer. Additionally, the introduced water reduces the viscosity of the alkaline solution and improves workability of the geopolymer mix. The content of Na_2_O and SiO_2_ in Geosil solution, optimized for geopolymer binders, makes it possible to avoid the need for adding sodium hydroxide. 

The mechanism of geopolymerization, setting of the geopolymer binder, consists of a sequence of successive reactions in a strictly defined order. When producing geopolymer materials, the sequence of activities is important. The correct introduction of the individual components guarantees the correct course of the required reactions. The process of preparing geopolymer mortars is carried out in stages. At the beginning, the alkaline solution was prepared. For this purpose, the total amount of water described in mortar composition was introduced into Geosil 34417 solution. In the first stage, the geopolymer binder consisting of fly ash, GGBFS, and alkaline solution was mixed. The binder mixing time was 10 min. From the chemical point of view, at the binder mixing stage the simplest, repeating structures are released which react with each other further in the synthesis process due to polycondensation reaction. As a result of the initiated processes, an extensive network structure is formed, which connects the geopolymer composite. Sand was added to the prepared binder. The whole thing was mixed for the next 3 min to obtain a geopolymer mortar with a high degree of uniformity. The mixing process was carried out in a laboratory mixer, adapted for preparing mortars. Table 3 shows the mortar compositions. All the components contained in the mixes are listed together with the density of individual materials. In addition, Table 3 provides the basic parameters describing the mixes. The values for the mortar contained in the table correspond to the percentage GGBFS content of the mix. The only parameter that differentiates mortar compositions is GGBFS content. As the mix compositions were given per 1 m^3^ of mortars, it was necessary to correct the content of the remaining components so that the same volume of the mix was provided for different amounts of fly ash and slag. Different FA and GGBFS densities affect the volumes of the prepared mixes. 

For mortar tests, 40 × 40 × 160 mm bar samples were prepared. Plastic molds with lids limiting the exchange of moisture with the environment were used. The setting of the mortars occurred at ambient temperature and humidity conditions of: T = 18 °C ± 2 °C, RH = 75%. No additional temperature treatment was applied. Samples with GGBFS were released from molds after 24 h, mortars without additive, based solely on FA—after 5 days due to the longer setting time of the mixes. The samples after mold release were stored under laboratory conditions (T = 18 °C ± 2 °C, RH = 75%). The applied testing procedure has a great influence on the assessment of the properties of the annealed material. To determine the effect of high temperature on the geopolymer mortar, after 90 days the samples were subjected to temperatures T = 200 °C, 400 °C, 600 °C, 800 °C and 1000 °C. The annealing process was carried out in an electric Nabertherm furnace equipped with an electronic system of annealing cycle programming. According to RILEM (Réunion Internationale des Laboratoires et Experts des Matériaux, systèmes de construction et ouvrages) recommendations, the temperature rise rate was 1 °C/min [36]. The low rate of temperature rise limits the thermal gradient between the outside and inside of the sample. Once the target temperature has been reached, the target temperature was maintained for two consecutive hours to ensure the most homogeneous temperature possible across the sample cross-section. Cooling took place spontaneously, inside the furnace chamber. In the further part of the article, the symbols according to Table 4 presented below are used.

The adopted test methodology allows the determination of the so-called properties of the material post exposure to high temperature. MIP porosity tests and microscopic observations were carried out on samples adapted to the requirements of testing equipment.

**Figure 1:**
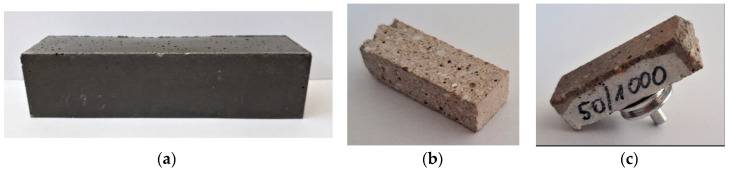
Samples for testing: (**a**) mortar beam 40 × 40 × 160 mm^3^; (**b**) sample for MIP test (**c**) sample for microscopic observations.

The test specimens were cut from 40 × 40 × 160 mm bars (Figure 1a) using a table saw in wet conditions. After cutting out, the elements had a cross-section of 1 cm^2^ and a height of about 3 cm. The materials were then cleaned under tap water and dried. The samples prepared in this way were given the effect of a thermal load. After the annealing cycle was completed, some of the samples were used for porosity testing (Figure 1b), while the rest was subjected to further preparations for microscopic observations. MIP tests and microstructural observations were carried out on single samples of each type of mortar. Porosimetry measurements were made using the mercury intrusion porosimetry method with the PoreMaster Automatic Pore Size Analyzer made by Quantachrome (USA).

Samples for microscopic examination had a regular shape and an observable surface of about 3 cm^2^. The surfaces on which the observations were carried out were hardened with resin and then sanded using sandpaper of various gradations. After grinding, the samples were rinsed in running water and polished to remove any traces of grinding. In the case of the reference samples, not exposed to high temperature, the grinding and polishing process was carried out wet with the use of water. To avoid the possible impact of water on changes in the structure of the material subjected to thermal loading, the surface preparation process of samples heated at 200 °C, 400 °C, 600 °C, 800 °C and 1000 °C was carried out in an anhydrous olive-based medium and no polishing was applied (Figure 1c). The microscopic observations were made with the Keyence VHX-7000 digital microscope and the Zeiss EVO-MA 10 scanning electron microscope using a BSD (backscattered electron detector) and EDS Bruker XFLASH 6/30 detector, under variable vacuum conditions.

## 3. Results

### 3.1. MIP Porosity

**Figure 2:**
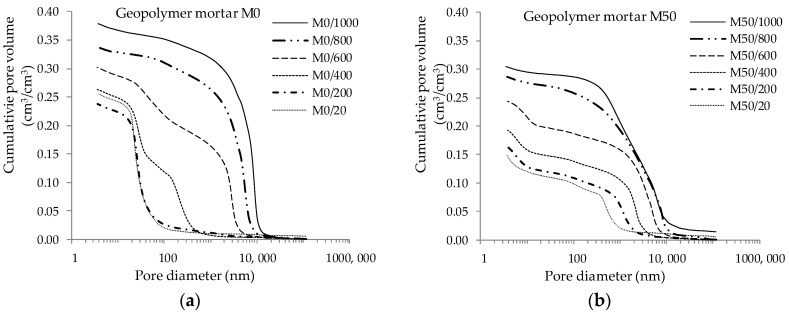
Total porosity: (**a**) mortars based on FA only; (**b**) mortar with GGBFS addition.

The total porosity of hardened geopolymer mortars determined after 90 days of curing (M0/20 and M50/20) is clearly different and depends on the GGBFS content in the mix. The test results are presented in Figure 2.

**Figure 3:**
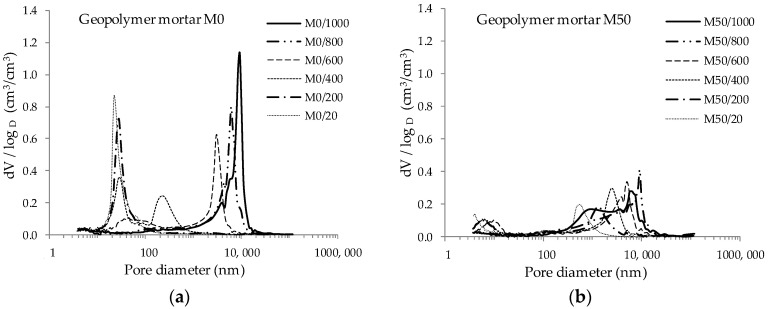
Pore distribution: (**a**) mortars based on FA only; (**b**) mortar with GGBFS addition.

The mortar with mixed FA-GGBFS binder shows porosity lower by about 40% (0.150 cm^3^/cm^3^) compared to mortar with FA only (0.240 cm^3^/cm^3^). The total porosity increases during the annealing process. In the case of mortar based solely on fly ash (M0), total porosity remains practically unchanged in the 20–400 °C temperature range. A minimal decrease in porosity was observed after exposure at 200 °C. Over 400 °C, porosity increases significantly with each successive temperature threshold. The mortar with GGBFS (M50) is also characterized by an increase in total porosity after exposure to high temperatures. The initially observed porosity difference between non-annealed materials of about 40% was gradually reduced after exposure to high temperatures. The observed porosity of M50 mortar was approx. 30%, 25%, 20%, 15% and 20% lower compared to the M0 mortar after exposing the materials to temperatures of 200 °C, 400 °C, 600 °C, 800 °C and 1000 °C respectively. The pore distribution presented in Figure 3 shows the differences in the internal structure for both materials.

**Figure 4:**
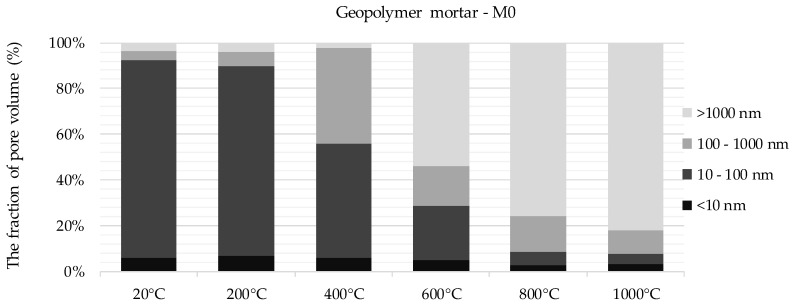
Categorization of pores for mortars based solely on FA (M0), depending on the temperature applied to the material.

**Figure 5:**
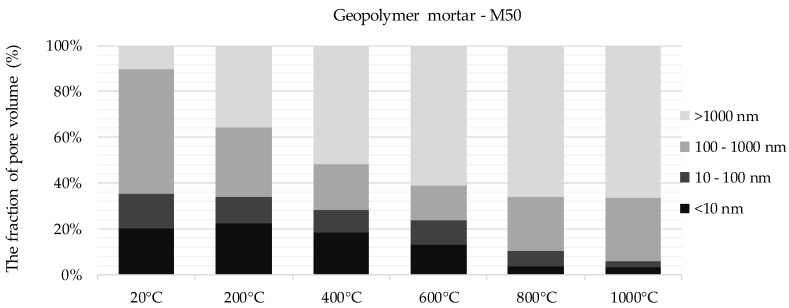
Categorization of pores for mortars with GGBFS (M50), depending on the temperature applied to the material.

The graph presenting the distribution of pores in M0 mortar shows clear peaks, indicating the dominant share of pores of certain sizes in the volume unit. Pores with small diameters dominate up to 400 °C. As the temperature increases, the proportion of pores with larger diameters increases, corresponding to changes in the microstructure of the material. This is particularly noticeable at temperatures in the range of 800–1000 °C. The curves showing the pore distribution for mortar with GGBFS (M50), due to the lower total pore volume, are characterized by a significantly lower proportion of pores of a given diameter per unit volume. To analyze more precisely the effect of temperature increase on porosity structure, the pores were categorized. Based on population curves, pores were divided into categories according to their diameter. The percentage of pores of a given diameter in a given category was then calculated. This presentation of the results enables a quantitative assessment of changes in the porosity structure of the tested materials as the temperature increases. The results obtained are presented in Figure 4 and Figure 5. 

The results of pore distribution analysis show that pores in the range from 10 to 100 nm predominate for non-annealed geopolymer mortar without GGBFS (M0). Pores with a diameter below 100 nm account for more than 90% of the total volume of the measured pores. Subjecting the material to high-temperature changes the share of individual pore categories in the overall material characteristics. After exposure at 200 °C, the changes were minimal. At 400 °C, a decrease in the proportion of pores between 10–100 nm and an increase in the presence of pores between 100 and 1000 nm was observed. However, the pores below 100 nm in diameter still represented more than 50% of the total porosity of the material. Observations made on the material after exposure to 600 °C indicate that pores with a diameter above 100 nm predominate. A very clear increase in the proportion of pores with the largest diameter was observed. Pores with a size above 1 µm started to exceed 50% of the total pore volume per unit of material volume. Up to 600 °C, the proportion of this category of pores did not exceed 5%. At subsequent stages of analysis after 800 °C and 1000 °C exposures, further increase in the share of pores with diameters above 1 µm was observed—to 75% and 82%, respectively. Changes in pore structure were also noticeable during microscopic observations. In the samples subjected to temperatures above 600 °C, a clear increase in cracking was observed both in the matrix and in the aggregate.

The pore structure of a non-annealed geopolymer mortar with mixed FA-GGBFS binder (M50) was dominated by pores in the range of 100–1000 µm—their proportion exceeded 50% of the total volume of the measured pores. Pores in the range of 10–100 nm accounted for 15% and pores with diameters below 10 nm for approx. 20% of total pore content per unit of material volume. Compared to mortars without GGBFS, the proportion of pores of the smallest diameter, below 10 nm, in the pore structure of mortar with mixed binder was more than three times higher. The analysis of the influence of high temperatures on the porosity of the material allowed to determine the characteristic directions of changes. Already above 200 °C, the share of pores with diameters above 1 µm increased to 35% and the share of pores with dimensions 100–1000 nm decreased to 30%. After exposure at 400 °C, pores with a diameter of 1 µm constituted already over 50% of the total porosity of the material. The share of the largest pores increased almost five times in relation to non-annealed material. The percentage share of pores with the smallest diameters, below 10 nm, was approx. 20% to 400 °C. Following exposure to 600 °C, the share of the smallest pores decreased significantly to 12%. Observations made on the material annealed at 800 °C and 1000 °C show a further increase in the proportion of the largest pores at the expense of a decreasing proportion of pores below 100 nm. After exposure to a temperature of 1000 °C, pores with diameters above 1 µm accounted for approx. 65% of the total pore content per unit of material volume. For comparison, in M0 material the share of pores from the described group exceeded 80%. The pore structure of the mortar with GGBFS was more varied compared to M0. The 100–1000 nm group constituted almost 30% and was almost three times higher than in M0 material after exposure at 1000 °C.

### 3.2. Microscopic Observations

**Figure 6:**
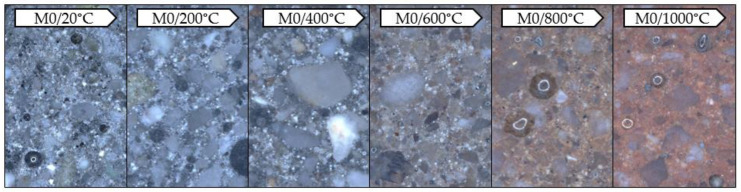
Photos taken with the Keyence VHX-7000 digital microscope, 50× magnification, mortar based on FA only.

**Figure 7:**
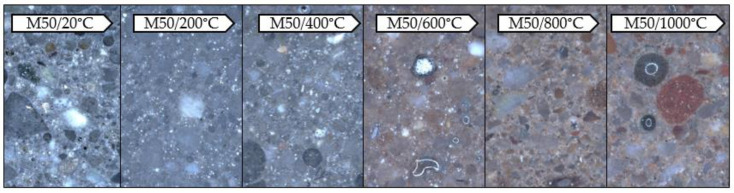
Photos taken with the Keyence VHX-7000 digital microscope, 50× magnification, mortar with GGBFS.

The observations were made with the digital microscope. The tests allowed to record the color of the binder subjected to exposition at a given temperature (Figure 6 and Figure 7). 

The samples subjected to temperature load processes have significantly changed their color depending on the temperature used compared to the reference samples (20 °C). This was observed both for the samples made exclusively from FA and those containing GGBFS. In both materials, a gradual change in color is visible as the applied temperature increases. This effect becomes more pronounced from approx. 600 °C. The color changes gradually from grey to rusty (copper). This is due to changes occurring in the material during the annealing process. The main factor causing the color change of the mortar when exposed to high temperatures is the chemical composition of the minerals used. The characteristic rusty color appearing at 600 °C is associated with the oxidation of iron compounds (mainly hematite Fe_2_O_3_) under the influence of temperature [37]. Due to the significantly higher content of iron oxide in FA (Fe_2_O_3_ = 6.32 wt.%) compared to GGBFS (Fe_2_O_3_ = 1.49 wt.%), the discolorations are more intense in a mortar based exclusively on FA (M0). 

**Figure 8:**
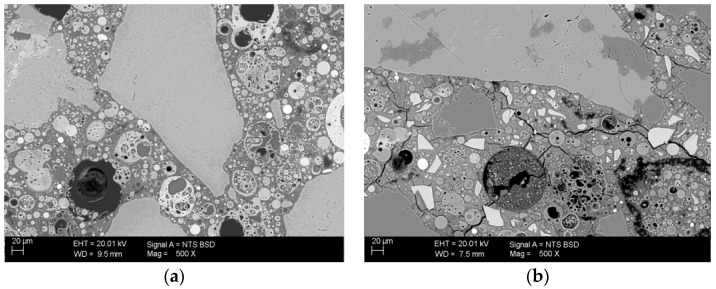
Photographs taken under a scanning microscope using a BSD detector, under variable vacuum conditions, magnification 500×, EHT 20kV: (**a**) mortar based on FA only (WD 9.5 mm); (**b**) mortar with GGBFS addition (WD 7.5 mm).

The same geopolymer samples were used to observe the microstructure in the scanning electron microscope using a BSD detector. The microstructure of both materials (M0 and M50) is clearly differentiated. These differences are already visible in samples not subjected to high temperatures. In addition to the composition of the binder, geopolymers differ significantly in the number of microcracks (Figure 8).

**Figure 9:**
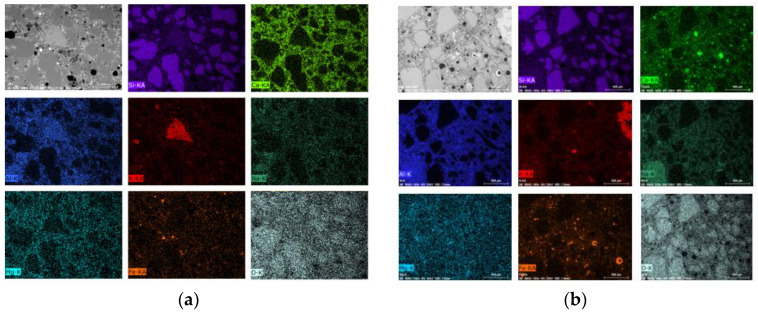
Mapping performed with a scanning microscope using an EDS detector, under variable vacuum conditions, magnification 100×, EHT 20kV: (**a**) mortar with GGBFS addition (WD 6.7 mm), M50/20 °C; (**b**) mortar based on FA only, M0/20 °C (WD 7.8 mm).

Sample M0/20 has a compact microstructure with virtually no cracking. On the other hand, the M50/20 sample with blast-furnace slag contains a large amount of microscopic material on the entire observed surface. Cracks in the sample with GGBFS are present in the entire volume, including the contact between zone grain and binder. The original cracks in this material are due to the use of slag as an additive. Its presence is demonstrated by calcium silicate grains (mainly belite), which are visible in the SEM image as white grains. Due to the presence of minerals typical of Portland cement clinker such as alite and belite, this material exhibits autogenous shrinkage during setting, resulting in the presence of cracks. The addition of slag results in a change in the chemical composition of the binder. It was presented by means of chemical analysis—mapping in selected micro-areas. Chemical analysis was carried out using the EDS Bruker XFLASH 6/30 detector. Mapping was performed on M0/20 °C and M50/20 °C reference samples (Figure 9).

The analysis was performed with marking typical elements for the matrix and aggregate. Purple is a marker of silicon, light green—calcium, navy blue—aluminum, red—potassium, dark green—sodium, blue—magnesium, orange—iron and light blue—oxygen. In a sample based solely on fly ash, more iron is observed than in a sample with slag, (this is shown in the form of orange numerous points) while the presence of calcium is at a much lower level (in the case of a GGBFS mortar, we observe calcium dispersed throughout the matrix). The contents of the other elements are at a comparable level. 

**Figure 10:**
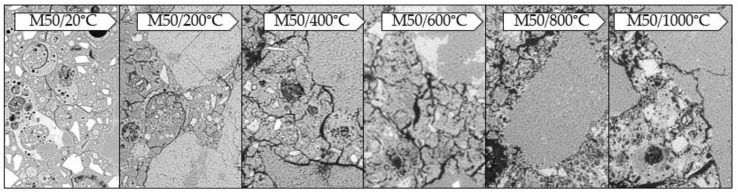
Photographs taken under a scanning microscope using a BSD detector, under variable vacuum conditions, 500× magnification, EHT 20 kV, WD 7.5–9.5 mm, mortar with GGBFS.

**Figure 11:**
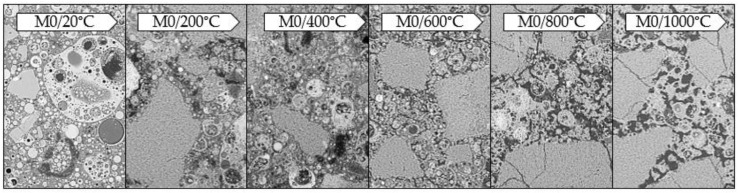
Photographs taken under a scanning microscope using a BSD detector, under variable vacuum conditions, 500× magnification, EHT 20 kV, WD 7.5–9.5 mm, mortar based on FA only.

As mentioned earlier, reference samples differ significantly in the degree of cracking. Figure 10 and Figure 11 show the change of the microstructure of the tested geopolymers depending on temperature exposure. It was observed that as the temperature increases, the degree of cracking also increases in both materials. 

By combining images obtained from a scanning microscope, it is possible to perform an analysis of the microstructure, taking into account the scratches that appeared in the material. The reference samples, based solely on FA, are virtually free of cracking. Cracks are very rare, and their width is below 1 µm. As the temperature affecting the material increases, the number and width of the microcracks increases as well. Cracks start to be more visible at 400 °C (below this temperature, there are practically no changes in the microstructure except for occasional microcracks on aggregate grains). The microcracks can reach a width of approx. 2–3 µm. They occur at the contact zone between the grains and the matrix, both in the matrix and the aggregate. From 600 °C onwards, however, many more cracks up to 5 µm in width appear. At 800 °C, we still see an increase in the number of cracks and the width of the resulting microcracks, which can reach up to 7 µm. The same is true at 1000 °C. The width of the microcracks reaches 10 µm and more. A similar relationship is observed for samples with GGBFS. As the temperature increases, the number and width of cracks increases; however, they are already present in the reference sample. In this case, we observe the microcracks that mainly occur at the contact zone between the grain and the matrix and within the matrix. Most numerous are cracks with the width of 1–2 µm, while the maximum width of the microcracks reaches 4 µm. At 200 °C, cracks also start to appear on aggregate grains. The number of microcracks increases significantly, with width reaching 5 µm. After exposure at 400 °C, the number of cracks increases even further, and their width reaches up to 10 µm. At 600 °C we observe a similar relationship, except that the width of the microcracks now increases to 12–13 µm, while at 1000 °C we observe microcracks as wide as approx. 20 µm. 

**Figure 12:**
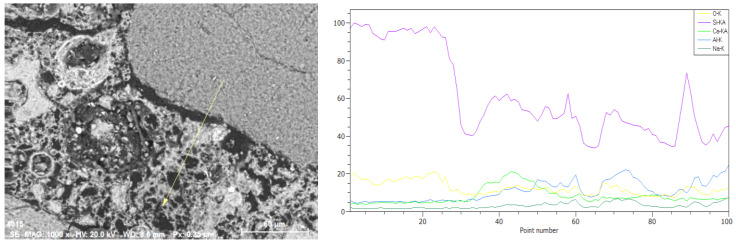
Linear analysis carried out with a scanning microscope using an EDS detector, under variable vacuum conditions, 1000× magnification, EHT 20kV, WD 8.6 mm, mortar with GGBFS annealed at 1000 °C, designated as M50/1000 °C.

**Figure 13:**
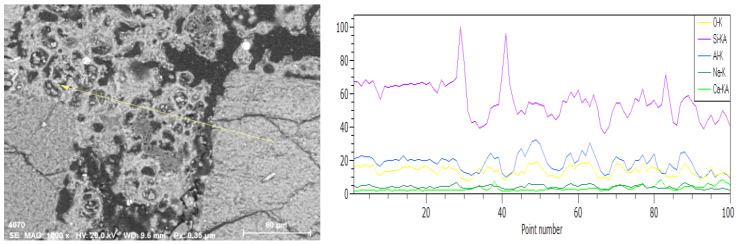
Linear analysis carried out with a scanning microscope using an EDS detector, under variable vacuum conditions, 1000× magnification, EHT 20 kV, WD 9.6 mm, mortar with FA only, annealed at 1000 °C, designated as M0/1000 °C.

In summary, the tendency of crack formation is similar in both materials. In both cases, therefore, the number of microcracks increases significantly with the temperature applied. They are formed in the matrix, at the interface between the grain and the matrix, and in grains. The matrix–grain contact zone seems to be the most sensitive and susceptible to micro cracking of the material, in both cases. To analyze this effect more precisely, a linear analysis was performed. The results for a geopolymer with slag (Figure 12) and one prepared solely based on fly ash (Figure 13) are presented below.

In both cases, a linear analysis was performed with the marking of characteristic elements for the matrix and inclusions. Yellow marking was used for oxygen, purple—silicon, light green—calcium, navy blue—aluminum and dark green—sodium. In samples subjected to temperature exposure, cracks can be observed at the grain-matrix contact zone, but also in the matrix itself and in the aggregate grains. However, the contact zone seems to be most weakened. The larger widths of the microcracks in this zone can be observed. They were filled with polymer during the preparation of the polished sections for testing. In addition to the cracks in the matrix itself, a higher porosity can also be observed than in the reference samples. 

## 4. Discussion

**Figure 14:**
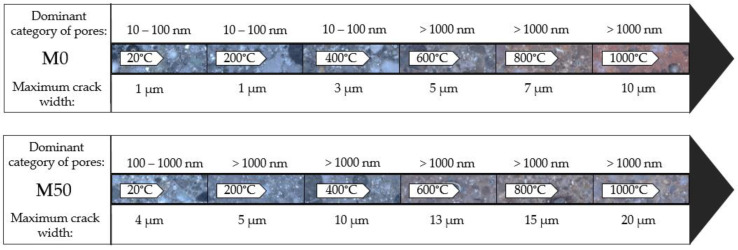
Diagram showing the most important changes in the microstructure of materials made solely based on FA and with the addition of GGBFS.

As mentioned in the introduction, in order to analyze the microstructure, MIP porosity test and microstructural observations in a scanning and digital microscope were performed. The first method provided information on the change in the porosity of geopolymers with changes in the temperature used, and also, thanks to the categorization of pores, made it possible to analyze the contribution of individual pore sizes. The microscopic observations confirmed changes in porosity, and also provided information on the microstructure and scratches in the material. Figure 14 presents the most important changes that occur in the material with the increase of the annealing temperature. The upper axis shows the characteristics of the mortar made on the basis of fly ash only, while the lower axis—mortar with the addition of slag. The axes describe the dominant category of pores and the maximum observed width of the cracks.

The observed cracks in reference material samples not exposed to high temperatures may be caused by chemical and physical phenomena accompanying the shrinkage of mortars. During the preparation of the samples, a clear effect of adding GGBFS on the rate of hardening of the material was observed. Mortar containing the mixed FA-GGBFS binder hardened much faster compared to mortars without GGBFS. The phenomena accompanying the setting of the material, resulting from shrinkage, could result in the damage of the microstructure of the material. Progressive chemical shrinkage while reducing plastic deformation due to rapid setting of the material may be one of the main causes of microcracks in non-annealed material. 

The number of cracks increases with increasing exposure temperature. The maximum width of scratches that could be measured during observation in a scanning microscope is also growing. The crack increase is caused by the influence of high temperature on geopolymers, which causes damage to the microstructure, visible as scratches. Under the influence of heating, the material loses water, its structure weakens and as a result, cracks appear.

Geopolymer mortar, similar to cementitious material, is a mineral composite for which changes in the microstructure at elevated temperature are caused by thermal incompatibility between the matrix and the inclusion, pore pressure effects and phase transformations [33]. Additional factors in the case of geopolymers are the effects of different raw materials [28], alkali cations [38] and calcium contents [39]. The observed decrease in porosity after exposure to the temperature of 200 °C results from the densification of the material structure. The temperature of 200 °C, which build a water vapor pressure not more than about 1.5 MPa, is far from the tensile strength of the geopolymer composite. Pore pressures cause minimal damage to the matrix. Inside the geopolymer structure, there are conditions which allow the formation of geopolymerization products. This products locate in pores in the microstructure. The effect of the decrease in porosity was seen for the sample without GGBFS addition with lower strength at room temperature [20]. According to the literature [40], geopolymers with lower strength at ambient temperature may retain more unreacted particles of raw materials, which, after creating appropriate hydrothermal conditions, can be transformed into geopolymerization reaction products. Up to a certain level, elevated temperature may determine a kind of temperature care, creating favorable conditions for the geopolymerization process. After temperatures above 200 °C, processes leading to the progressive destruction of the material structure begin to dominate. The geopolymer microstructure under high temperature is enhanced by the progressive geopolymerization of unreacted precursor particles. Structural damage results from thermal incompatibility and is also a function of the ductility level of the material. The dominant processes in a given temperature range are responsible for the form of the geoplymer microstructure [41].

## 5. Conclusions

Both materials (M0 and M50) have an amorphous structure regardless of the annealing process.. Furthermore, materials made both on the basis of FA and GGBFS show a stability of chemical composition. As the temperature increases, the number of cracks increases, together with porosity, as demonstrated in the SEM images. This is also confirmed by the MIP porosity test, which shows how the pore distribution changes in the samples. In the geopolymer based solely on FA, over 90% of the pores in the reference sample have a diameter of less than 100 nm. As the annealing temperature changes, the number of pores with a diameter between 10–100 nm decreases, while the number of pores with a diameter greater than 1000 nm increases. This is slightly different for a geopolymer with a mixed FA-GGBFS binder. In this case, the reference sample has the largest number of pores with a diameter between 100 and 1000 mm. As the temperature changes, the number of pores over 1000 nm increases. Nevertheless, there are still 15% less of such pores than in the sample based solely on FA. The width of cracks also increased with the value of the temperature used for annealing. For the M0 sample at 1000 °C, cracks up to approx. 10 µm appeared, and for the M50 sample–even over 20 µm at the same temperature of annealing. The microstructure of materials is amorphous, and as the annealing temperature increases, the individual elements (grains, fly ash microspheres) become less and less visible. No sharp, pronounced connections can be observed at the phase boundary, which could be clearly observed in samples not subjected to high temperatures. In addition, the color of geopolymers changes significantly when the temperature rises above 600 °C. The appearance of characteristic rusty discoloration results from the oxidation of iron compounds under the influence of temperature. Fly ash has about 4 times the iron oxide (Fe_2_O_3_) content compared to GGBFS, hence the more pronounced color change in the case of a mortar based solely on FA (M0). The increase in porosity and cracks as the annealing temperature increases, will certainly affect the results of other studies in the macro scale. 

Material based solely on FA achieves smaller crack widths than with the addition of granular blast-furnace slag (GGBFS). Their frequency of occurrence is also lower. Therefore, it can be concluded that it is more resistant to the effects of high temperatures. Despite the slight differences, both materials behave similarly.

The rest of the executed research plan provides for the extension of microstructural studies. These tests will be aimed at identifying the phenomena occurring in the heated material. For this purpose, the crystal structure and phase composition by X-ray diffraction (XRD) will be determined, and chemical bond analysis will be performed using infrared spectroscopy. In addition, the mechanical properties of materials subjected to high-temperature exposure will be determined.

## Figures and Tables

**Table 1:** Oxide composition of the fly ash (FA) and ground granulated blast-furnace slag (GGBFS) in wt.%, data supplied by the manufacturer.

(wt.%)	SiO₂	Al₂O₃	Fe₂O₃	CaO	MgO	SO₃	K₂O	Na₂O	P₂O_5_	TiO₂	Mn_3_O_4_	Cl^−^
FA	52.30	28.05	6.32	3.05	1.71	0.28	2.51	0.76	0.69	1.35	0.07	-
GGBFS	39.31	7.61	1.49	43.90	4.15	0.51	0.36	0.47	-	-	-	0.038

**Table 2:** Geosil—product characteristics. Based on data provided by the manufacturer.

Characteristic	Woellner Geosil 34417	Unit
Na_2_O content	16.74	wt.%
SiO_2_ content	27.5	wt.%
density	1.552	g/cm^3^
viscosity	470	mPa·s
weight ratio (WR = wt.% SiO_2_/wt.% Na_2_O)	1.64	-
molar ratio (MR = mol SiO_2_/molNa_2_O)	1.70	-

**Table 3:** Geopolymer mix compositions and basic mortar parameters.

Components	ρ	M0	M50
	(g/cm^3^)	(kg/m^3^)	(kg/m^3^)
Alkaline solution (Geosil 34417 + extra water)	1.41	330.7	347.5
FA	2.10	734.9	386.1
GGBFS	2.90	0.0	386.1
Sand (0/2 mm)	2.65	1102.4	1158.3
**Mortar parameters**			
sand to binder weight ratio (sand/FA + GGBFS)		1.5	1.5
alkaline solution to binder (FA + GGBFS) weight ratio		0.45	0.45
water to binder (FA + GGBFS) weight ratio		0.3	0.3

**Table 4:** Designation of geopolymer samples.

Specimen ID	FA Addition	GGBFS Addition	Exposure Temperature
M0/20	+	-	Room temperature
M0/200	+	-	200 °C
M0/400	+	-	400 °C
M0/600	+	-	600 °C
M0/800	+	-	800 °C
M0/1000	+	-	1000 °C
M50/20	+	+	Room temperature
M50/200	+	+	200 °C
M50/400	+	+	400 °C
M50/600	+	+	600 °C
M50/800	+	+	800 °C
M50/1000	+	+	1000 °C
